# Evaluation of Machine Learning Classification Models for False-Positive Reduction in Prostate Cancer Detection Using MRI Data

**DOI:** 10.3390/diagnostics14151677

**Published:** 2024-08-02

**Authors:** Malte Rippa, Ruben Schulze, Georgia Kenyon, Marian Himstedt, Maciej Kwiatkowski, Rainer Grobholz, Stephen Wyler, Alexander Cornelius, Sebastian Schindera, Felice Burn

**Affiliations:** 1 Institute for Medical Informatics, University of Lübeck, 23562 Lübeck, Germany; marian.himstedt@uni-luebeck.de; 2 Fuse-AI GmbH, 20457 Hamburg, Germany; ruben.schulze@fuse-ai.de; 3 Australian Institute of Machine Learning, University of Adelaide, Adelaide, SA 5005, Australia; georgia.kenyon@adelaide.edu.au; 4 Precision Imaging Beacon, University of Nottingham, Nottingham NG7 2RD, UK; 5 Department of Urology, Cantonal Hospital Aarau, 5001 Aarau, Switzerland; 6 Medical Faculty, University Hospital Basel, 4056 Basel, Switzerland; 7 Department of Urology, Academic Hospital Braunschweig, 38126 Brunswick, Germany; 8 Institute of Pathology, Cantonal Hospital Aarau, 5001 Aarau, Switzerland; 9 Medical Faculty, University of Zurich, 8032 Zurich, Switzerland; 10 Institute of Radiology, Cantonal Hospital Aarau, 5001 Aarau, Switzerlandfelice.burn@ksa.ch (F.B.); 11 AI & Data Science CoE, Cantonal Hospital Aarau, 5001 Aarau, Switzerland

**Keywords:** machine learning, deep learning, multiparametric MRI, medical imaging, prostate cancer

## Abstract

In this work, several machine learning (ML) algorithms, both classical ML and modern deep learning, were investigated for their ability to improve the performance of a pipeline for the segmentation and classification of prostate lesions using MRI data. The algorithms were used to perform a binary classification of benign and malignant tissue visible in MRI sequences. The model choices include support vector machines (SVMs), random decision forests (RDFs), and multi-layer perceptrons (MLPs), along with radiomic features that are reduced by applying PCA or mRMR feature selection. Modern CNN-based architectures, such as ConvNeXt, ConvNet, and ResNet, were also evaluated in various setups, including transfer learning. To optimize the performance, different approaches were compared and applied to whole images, as well as gland, peripheral zone (PZ), and lesion segmentations. The contribution of this study is an investigation of several ML approaches regarding their performance in prostate cancer (PCa) diagnosis algorithms. This work delivers insights into the applicability of different approaches for this context based on an exhaustive examination. The outcome is a recommendation or preference for which machine learning model or family of models is best suited to optimize an existing pipeline when the model is applied as an upstream filter.

## 1. Introduction

According to the latest epidemiological data, prostate cancer (PCa) is the most frequently diagnosed cancer among men in 112 countries worldwide [1]. In 2022, 397,430 PCa-related deaths and 1,467,854 newly diagnosed PCa cases were reported worldwide [2], making PCa the eighth leading cause of cancer death in both sexes [2]. An increasing trend is expected as the population is aging, and age and risk of PCa are correlated [3]. In the case of early detection of PCa, curative treatment is possible, which causes a reduction in mortality. Multiparametric MRI (mpMRI) of the prostate plays an important role in the diagnostic pathway for the early detection of PCa. This is where computer-aided diagnosis can be utilized. Artificial intelligence (AI) has gained popularity in many fields, including medicine. Kufel et al. [4] provide a detailed introduction to AI and machine learning (ML) in medicine and highlight common challenges, such as the scarcity of labeled training data that would be needed to train a reliable classifier with distinctive generalization capabilities. Once trained successfully, AI can aid in the automation of intellectual tasks that are regularly performed by humans. However, the clinical adoption and validation of AI-based systems need to be carried out carefully since ML models may only predict true conditions within certain limits [4].

The current state-of-the-art medical AI solutions report AUC-ROC scores of 0.8328 ± 0.0878 for lesion detection and characterization [5]. These approaches often only cover the evaluation of the classifier as a standalone with known lesion locations but do not cover the big picture of lesion classification together with localization as a complete pipeline. In real-world applications, it can be necessary to construct holistic systems that locate and classify potentially malignant lesions. Such end-to-end pipelines exist in the literature [6,7] or as certified medical products developed by FUSE-AI GmbH in Hamburg, Germany. The architecture of such products is commonly based on a sequential pipeline utilizing one or more UNet models to predict the location of certain anatomies of the prostate. The FUSE-AI pipeline uses mpMRI sequences as input and predicts the segmentations of the prostate gland and its zones. It proceeds to generate voxel-wise lesion location predictions together with a label, i.e., background, benign, or malignant. These holistic solutions are often prone to delivering overly sensitive predictions, resulting in higher false-positive rates. This could possibly cause unnecessary surgery, e.g., prostate biopsy in the case of a false-positive finding. Therefore, there is a need for a reduction in the false-positive rate, i.e., an increase in the specificity with maintained or improved sensitivity.

**Figure 1:**
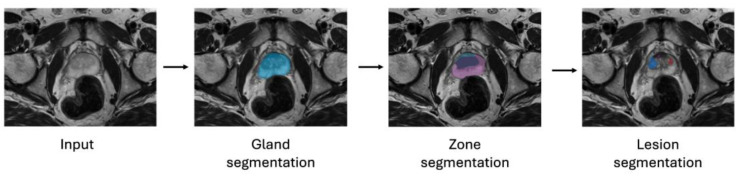
Simplified setup of a sequential lesion segmentation/classification pipeline.

In this work, several ML approaches are evaluated in terms of their ability to improve the lesion classification performance of the UNet-based pipeline, as shown in Figure 1. It is not desired to propose an entirely new method or apply broad changes to the pipeline but rather to analyze the impact of state-of-the-art methods on an existing pipeline with the overall aim of improving its performance for false-positive reduction in prostate cancer detection.

However, during evaluation, it is best to use as little segmentation/localization as possible. A strong classifier that does not rely on voxel-wise annotations could have several advantages: (1) it benefits from a larger amount of data, as image-level labels are easier to obtain, resulting in a more robust and stable classifier; (2) it offers an immediate warning for extra attention if the case appears to contain malignant lesions, even before the image is reviewed by a radiologist; and (3) it can be used as an upstream filter without the need to run computationally expensive segmentation models. A robust and reliable whole-image classifier could save time and resources by minimizing unnecessary segmentation model inferences for MRI scans that do not contain PCa.

This study focuses on evaluating the tendency of different ML models best suited for this problem, considering how the performance is influenced by the inclusion of localization information and whether classical machine learning or deep learning is more beneficial. The emphasis of this study is on the process of model evaluation rather than model development.

Three main questions emerge that will be covered in this work:

- Is the classification performance affected by incorporating localization information using the ROI?
- Is classical machine learning or deep learning more beneficial in solving the issue?
- Can the pipeline performance be improved by adding a classification algorithm?

To assess these main questions, various well-established machine learning methods are evaluated using carefully chosen data, as detailed in Section 2. The results are presented in Section 3, with subsections comparing whole-image classification to region-based classification.

This paper then provides an extensive discussion of the results in Section 4, comparing the achieved results to the state of the art and highlighting the study’s limitations. Finally, a brief conclusion is provided, answering the three main questions posed earlier.

## 2. Materials and Methods

### 2.1. Image Data and Labels

A total of 1166 cases from one public dataset (ProstateX [8], *n* = 182) and two private datasets were retrospectively collected. The private datasets were provided to FUSE-AI GmbH by Kantonsspital Aarau (Aarau, Switzerland) (*n* = 895), and Universitätsklinikum Jena (Jena, Germany) (*n* = 89). All data sources used SIEMENS Skyra 3.0T MRI devices for the image acquisition. Each image was normalized and then resampled to a voxel spacing of 0.75, 0.75, and 3 and cropped/padded to a size of 149 × 149 × 32 voxels, using the transverse views of the T2-weighted (T2W), apparent diffusion constant (ADC), and diffusion-weighted images (DWI) sequence.

Cases containing a T2W image of insufficient quality due to artifacts that obscure the prostate in the image, such as those caused by endorectal coils, hip implants, or anatomical phenomena (e.g., bladder protruding into the prostate or similar), were excluded from the dataset. Patients were included if they were male and suspected of having prostate cancer.

Cases for patients meeting any of the following criteria were excluded from the dataset:

- Patients younger than 45 years;
- Patients who had undergone previous prostate surgeries (ICD-10-PCS 0VT0 Resection of Prostate, 0VB0 Excision of the Prostate);
- Patients (ICD 0VB-03ZX) who had a prostate biopsy less than six months ago.

Only the transverse view of all series was used; coronal and sagittal views, if present, were discarded.

Labels are based on the Gleason score, which provides reliable information about the condition of the tissue. According to the scoring system definition [9], a Gleason score of >3 + 3 = 6 is considered clinically significant. Therefore, the region of interest (ROI) that includes the lesion was labeled with a 1 for analysis purposes. To indicate clinically insignificant (benign) lesions or no lesions at all, a study label of 0 was assigned. This labeling was achieved by mapping histopathological report findings to the image.

Due to inconsistencies, errors, and incompleteness in human annotation, the ground truth was found to be of insufficient quality, and the entire study, including the image data, was excluded from the dataset.

In total, 718 benign and 448 malignant cases were included. The ROI can cover the entire image or certain anatomies within the image.

All cases included in the final dataset were fully anonymized and explicitly cleared for research (and/or commercial) use; therefore, no further ethics assessment was conducted.

### 2.2. Models

For classical ML, we used a support vector machine (SVM), multilayer perceptron (MLP), and random decision forest (RDF), specifically XGBoost (referred to as XGB). The hyperparameters for each of the classifiers were optimized as described in Section 2.3. In addition to classical ML methods, we investigated convolutional neural networks (ConvNet, ConvNeXt, and ResNet). A standard ConvNet was implemented, consisting of several blocks, each containing two convolutional layers followed by a MaxPooling layer with a pooling size of (2, 2, 2). The output feature map is pooled using global average pooling and classified using a linear layer and sigmoid output activation. The model has a total of 168,705 trainable parameters when using the smallest layout with two blocks. The second model utilized model was a residual network (ResNet) [10], implemented with eight convolutional blocks. Each block consists of a convolutional layer, a batch normalization, and a ReLU layer, resulting in a total of 4,527,906 trainable parameters. Recently, Liu et al. introduced a novel approach to further improve the ResNet by incorporating principles from vision transformers [11]. In the final 3D adaptation for this model, 31,321,561 parameters needed to be trained. All models were adapted to process anisotropic 3D data by replacing 2D convolution and pooling blocks with 3D convolution and pooling blocks, using respective anisotropic kernels. To evaluate a pure transfer learning approach, we utilized the architecture and CT pretrained weights from Models Genesis [12]. Models Genesis describes a self-supervised learning framework for deep models to learn common anatomical representations automatically. To enable the 3D models to learn a transferable image representation, the authors used sub-volumes of the original image (chest CT), deforming them so the model learns to restore the original sub-volume. 

For this work, the weights and UNet architecture from lung cancer detection on CT data (Models Genesis) were applied and fitted to the prostate MRI data. Additionally, we evaluate the training of a UNet as autoencoder (AE) for input compression in combination with a Softmax classifier.

All models output either an integer label (0 or 1) or a probability vector that can be converted to a label of 0 or 1, indicating whether the analyzed region is likely to contain a malignant lesion (1) or not (0).

### 2.3. Methods

For classical ML, performance on the whole image was assessed using different combinations of feature sets and classifiers. The models required textural features, represented by Haralick features, 3D implementations of local binary pattern (LBP) [13], and histogram of oriented gradients (HOG) [14]. A total of 297 features per sequence were extracted, resulting in a total of 891 features when using T2W, ADC, and DWI. The maximum relevance—minimum redundancy (mRMR) feature-selection method [15], as well as the principal component analysis (PCA), were used. Additionally, SMAC3 hyperparameter tuning [16] was applied to optimize hyperparameters for the classical ML classifiers. CNN-based experiments on the whole image were carried out by incorporating techniques such as hyperparameter tuning, regularization, data augmentation, dropout, early stopping, and weighted losses. Convolutional autoencoders were used for the creation of an embedding to be classified by a softmax classifier. Anomaly detection was also investigated following [17]. Transfer learning approaches, such as transferring weights from segmentation models to classification models or using weights from Models Genesis to MRI tasks, were considered. Furthermore, a Jigsaw-puzzle-solving pretraining task was evaluated, where the model theoretically learns the image composition by ordering a sequence of permutations of systematically chosen image patches, consolidating methods proposed in [18,19].

To investigate the effect of incorporating localization information, various region-based classification approaches were implemented. Region-based approaches use the same models as whole image approaches but use bounding boxes around certain anatomical regions as input instead. The regions used included the prostate gland, the peripheral zone (PZ), and lesions, with each subsequent region gradually decreasing in size and increasing in localization information. During training, the anatomical regions were known from ground truth segmentations, and during inference, the pipeline models predicted the segmentation of the required anatomy. Reducing the input space to the gland size was expected to enhance the performance compared to the whole-image classification by minimizing background noise. Given that 70% of prostate lesions are found in the PZ [20], the reduction in the input space to the PZ was expected to be particularly beneficial. The tumors in the PZ are best seen on the ADC sequence [21], so only the ADC sequence was used as input for the analysis. Bounding boxes around known lesion locations were also tested, as suggested by [5].

A sliding window approach is useful when classifying small regions of an image without providing spatial information for ROI extraction during inference [22]. Therefore, a window of size 70, 65, and 3 voxels (x, y, and z directions, respectively) was moved across the image. The true label for the window content was determined by its overlap with a lesion. If the window/lesion over-lap ratio exceeded 0.5, the window was labeled with the lesion’s label (benign or malignant); otherwise, it was labeled as background.

Due to the convolutional and pooling layers of the CNNs, the sliding window size was adjusted to 64x-64x-8 voxels for CNN-based approaches. The windows overlapped by 50% of their width. To obtain an image-based classification, the confidence scores of each window were used to generate a heatmap, highlighting areas likely to contain potentially malignant lesions. The heatmap was thresholded at a specific cut-off point determined from the ROC curve. If areas in the heatmap exceeded this confidence threshold, the image-level label was set to malignant; otherwise, it was assigned as benign.

### 2.4. Experiment Setup

All classifiers were first evaluated as standalone classifiers on the whole image as well as in region-based setups. To increase robustness, the performance metrics were averaged using 5-fold cross-validation. An 85/15 training/test split of the data was made, with 85% used for training (divided into the five folds) and 15% used as the test set. 

**Figure 2:**
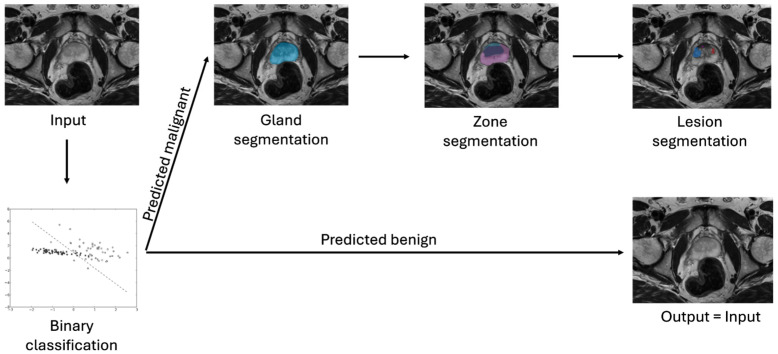
Pipeline evaluation mode 1: All classifiers that do not require lesion segmentations are placed at the beginning of the pipeline or after the model that predicts the required anatomy.

**Figure 3:**
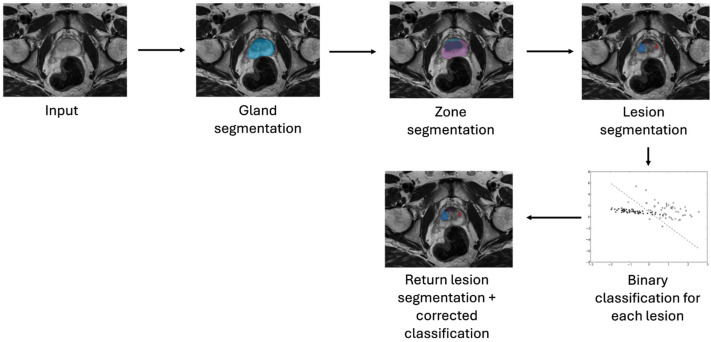
Pipeline evaluation mode 2: The lesion classifiers are placed at the end of the regular pipeline to produce a classification of the lesion candidates selected by the existing pipeline segmentation models.

After standalone training, the classifiers were combined with the lesion segmentation pipeline to evaluate their impact. Depending on the required input (whole image, gland, zone, or lesion), the classifier was positioned in the pipeline accordingly and triggered different mechanisms. The whole image, gland, and zone classifiers filtered out all images predicted to be lesion-free, omitting the inference of segmentation models for those images, as depicted in Figure 2. The lesion-based models used the segmentations proposed by the pipeline and assigned a label to each segmented lesion, as shown in Figure 3. To measure and compare the performance of the classifiers, both as standalone and in combination with the pipeline, the following metrics were used: Area under the Curve of the Receiver Operating Characteristic (AUC-ROC), sensitivity, and specificity. In these metrics, the positive class was the malignant class.

## 3. Results

### 3.1. Whole-Image Results

The results of the classical ML image classifiers are presented in Table 1. The approaches were evaluated in different settings, achieving up to 0.6016 AUC-ROC on the test partition when using the whole image.

The different feature-extraction and feature-selection methods did not significantly impact the performance, nor did the SMAC3 hyperparameter optimization for any of the classifiers. SMAC3 evaluated the best parameter setting for each classifier, optimizing parameters such as the regularization parameter, kernel function, or class weight for the SVM. XGBoost was optimized by the learning rate, maximum depth of a tree, and the maximum number of leaf nodes. For the MLP, various numbers of layers and layer sizes were tested. During the experiments, XGB and SVM performed similarly both on the training partition and on the test partition compared to the MLP. The best overall performance was achieved by the SVM classifier, with the error tolerance parameter set to 60, using 95 principal components returned from PCA on LBP, HOG, and Haralick features. The standard ConvNet, ConvNeXt, and ResNet, in combination with standard optimization methods, returned AUC scores of about 0.5273 ± 0.0238. Table 2 shows that the best performance for the standard models was achieved by the ConvNeXt architecture, yielding a 0.5038 AUC-ROC. To improve the performance, pretraining and transfer learning were applied.

**Figure 4:**
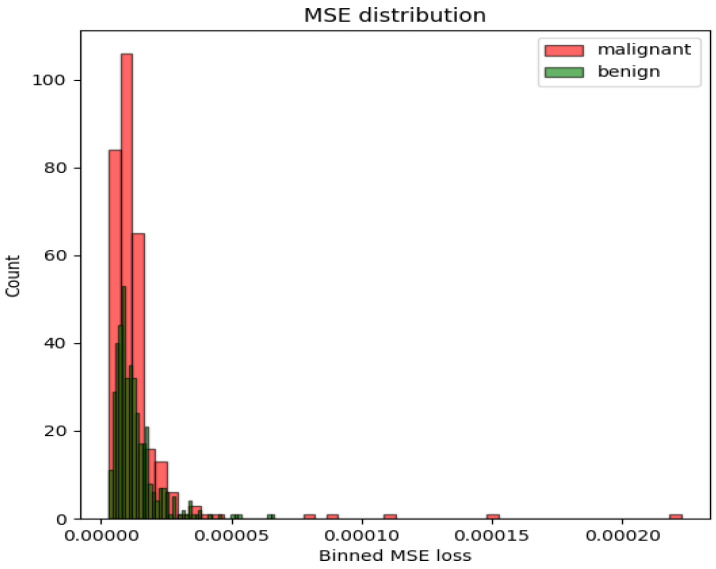
MSE loss distribution with respect to the two classes.

The Jigsaw-puzzle-solving approach did not deliver the anticipated performance improvement. However, applying an AE based on the ConvNeXt architecture (two mirrored ConvNeXt models comprising a UNet-like architecture) to create an embedding to be classified by a softmax classifier yielded the best results for the whole image with an AUC-ROC of 0.6553. The Genesis model, fine-tuned with MRI data using its CT pretrained weights, performed better than the plain CNN-based architectures but slightly worse than the AE. Using an autoencoder for anomaly detection by measuring the reconstruction Mean Squared Error (MSE) of images from different classes resulted in a random classifier, as no difference between the classes could be determined, as shown in Figure 4. The variational autoencoder yielded similar reconstruction probabilities from the latent space for benign and malignant images, preventing any distinction either.

**Figure 5:**
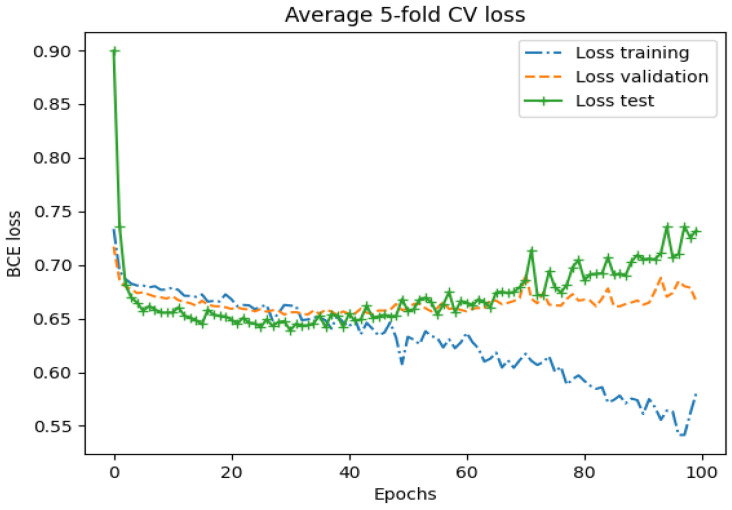
Average 5-fold cross validation loss (binary cross entropy loss) for the training, validation, and test partition on the whole image with the Genesis model over 100 epochs.

**Figure 6:**
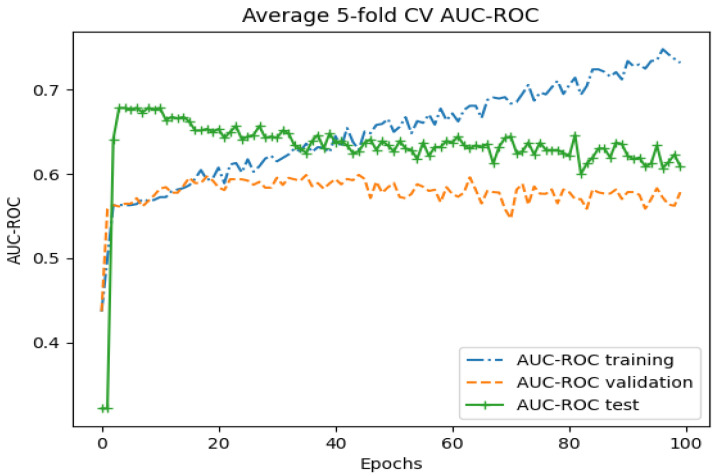
Average 5-fold cross-validation AUC-ROC for the training, validation, and test partition on the whole image with the Genesis model over 100 epochs.

Averaging the five folds of the training and validation of the whole-image classification with the Genesis model indicated that the model was prone to overfitting, as shown in Figure 5 and Figure 6. A diverging AUC-ROC score and binary cross-entropy (BCE) were observed after around 40 epochs (approximately 10,000 optimizer steps). Countermeasures such as dropout, regularization, and reduced model complexity did not show improvement. 

### 3.2. Region-Based Results

Assessing the previously established methods on regions to introduce a stronger localization showed mixed results. Table 3 presents the best results for each region-based approach, along with the best-performing classifier for that region. While the SVM delivered the strongest results for whole-image classification in the area of the classical ML, the XGB classifier yielded the relatively best scores by a small margin across all regions. Using a segmentation of the prostate gland delivered no improvement compared to the classification of the whole image. The PZ setup led to significantly decreased scores compared to the whole-image classification approach. However, using lesion segmentations achieved the best performance, with an AUC-ROC of 0.6897. The sliding-window approach with classical ML classifiers delivered results close to random chance. For CNN-based methods, only the best-performing approaches (AE and Genesis) were compared. In Table 3, it can be observed that the Genesis model yielded the best scores for all regions. The Genesis model outperformed the AE by at least 10 percentage points in the AUC-ROC. Additionally, performance on the gland segmentation with CNN-based models did not improve by using a bounding box around the gland when compared to whole-image classification.

Classifying the PZ decreased the performance on both the test partition and the training partition. Using lesion segmentation as input provided reasonable performance when transfer learning with the Genesis model. However, the sliding-window approach did not deliver the anticipated results as a standalone classifier.

### 3.3. Performance Measurement in the Segmentation Pipeline

The goal of this work is to reduce the false positives in a lesion segmentation pipeline. To measure the impact of the aforementioned classifiers, the best performing models were integrated into such a pipeline. Table 4 shows that none of the classifiers was able to achieve outstanding improvements compared to the baseline. Interestingly, the best results in the pipeline were not necessarily achieved by the best standalone classifiers. Overall, only the Genesis-based sliding window approach improved specificity by 0.0225 while maintaining the sensitivity.

Similar to the graphs presented in Figure 4 and Figure 5, the Genesis model overfitted the training data, yielding perfect scores on the training partition. Applying countermeasures such as dropout, regularization, and reduced model complexity did not prevent the model from overfitting. However, the validation and test results were improved compared to the whole-image results.

## 4. Discussion

### 4.1. Discussion of Methods

The applied methods demonstrated the potential to improve the pipeline performance, but only one approach was beneficial. Most methods resulted in unbalanced metric values, indicating room for improvement. Exhaustive research of all parameter combinations was unfeasible due to the extensive range of investigated methods and models investigated. Minor changes might lead to better performance in the pipeline. Additionally, the methods based on the whole image, instead of lesion segmentations as originally proposed, likely compromised the performance. For example, Min et al. [15] selected 819 radiomics features from lesion segmentations and reduced the feature set to a total of 9 features by using mRMR and LASSO. The authors reported an AUC-ROC of 0.823, sensitivity of 0.841, and specificity of 0.727 (test dataset) when classifying csPCa vs. non-csPCa. In this work, applying the method to the whole image returned 0.5461 AUC-ROC, 0.5000 sensitivity, and 0.5921 specificity. Even when applied to lesion segmentations, Min et al.’s results could not be matched, achieving an AUC-ROC of 0.6768 with the SVM. The best result using a radiomic feature set and classical machine learning methods on lesion segmentations was an AUC-ROC of 0.6897 with a sensitivity of 0.8462 and 0.5333 specificity, obtained with an XGB model. This demonstrates that lesion-based methods using the whole image returned worse results compared to the results obtained with the intended use. Feature selection plays an important role, as Min et al. achieved higher scores with different feature compositions. The feature set used in this study could be improved by using different feature-extraction methods, such as stacked sparse autoencoders for dynamic feature selection, as proposed in [23], which perform better than PCA. 

For zone-based classification, it could be beneficial to include only one specific subset of features, as proposed in [24]. The authors reported a high AUC-ROC of 0.92 in the PZ by using a total of 26 features, identified using a semi-exhaustive search to find the best feature composition to identify lesions within the PZ. In this study, the best score for zone-based classification was 0.5467 AUC-ROC using texture features. The number of features was regulated by applying PCA or mRMR, differing from the semi-exhaustive search. Better performance might be achieved by selecting a feature subset using a more effective sampling method.

The lesion-based classification has been reported with high scores with state-of-the-art methods. For instance, an AUC-ROC score of 0.95 for the binary classification of csPCa vs. non-csPCa was reported using 1281 radiomic features [25]. In this work, the highest AUC-ROC for lesion classification using classical machine learning was 0.6897. The lower scores may be due to the feature-extraction approach. The chosen extraction methods were originally designed and applied for 2D problems but were adapted to 3D for this study, potentially comprising their performance and leading to lower AUC-ROC scores compared to the state of the art. Common methods rely on 2.5D approaches to analyze 3D volumes with 2D feature extraction methods. However, 2.5D approaches were not utilized in this study and should be investigated in the future.

For localization-free classification, a sliding-window approach was chosen. A window was moved over the image to produce a heatmap of probabilities for malignant lesions following the method described in [18]. The applied sliding-window approach offered low localization performance, as the model favored lesion-like objects outside the prostate. Future approaches should incorporate further restrictions to limit findings only to the prostate gland.

Previous work reported 0.73 to 0.79 accuracy, sensitivity, and specificity using sliding windows by employing a tumor-candidate-detection model to propose regions for further classification [26]. By extracting various features (intensity features, first- and second-order statistics, region sizes, morphologic features, asymmetry features, and physiologic features), strong classification was realized. Despite this setup closely resembling the second pipeline evaluation mode presented in this work, the authors extracted different features that better represented the data. In contrast, the features extracted during the experiments could not represent the data well enough to grant strong classification performance.

A multiscale sliding window approach could also improve performance, achieving up to 0.96 sensitivity at 0.95 specificity [22]. For this purpose, voxel-wise classification was created by classifying textural features and incorporating clinical information such as the location and shape of the lesion. The absence of clinical features may account for the lower results achieved in this work. Furthermore, no voxel-wise classification was carried out due to the high computational effort. Both of the state-of-the-art sliding-window approaches [22,26] used relatively small window sizes and based their feature extraction on 2D windows instead of 3D cubical windows. Multiscale and 2.5D approaches were employed in state-of-the-art studies for both classical machine learning and deep learning [25,27]. In this work, the sliding-window approach to the whole image was utilized with a small z dimension, resembling a 2.5D approach. The obtained results led to an improvement in the pipeline performance. Considering that reformulating and solving tasks in 2D loses rich 3D anatomical information, which can comprise the model [12], a true 2.5D approach was not chosen to maintain the depth information contained in the 3D image. However, it is possible that the pipeline performance could be increased by adhering to a 2.5D approach using a modified ConvNet presented in [27]. The authors applied an XmasNet to classify modified 2D slices of ROIs around the lesion, yielding 0.95 AUC-ROC; however, due to time constraints, this method was not investigated in this work.

Different approaches were chosen for pretraining the CNN models. The Jigsaw puzzle-solver model, in particular, did not perform well in detecting permutations and thus did not benefit the PCa-classification task. It is possible that the task was overly complex for a 3D volume. Originally, the approach was used for the detection of large objects on natural two-dimensional images. There are adaptations to 3D [28] where the task was simplified. Instead of determining a permutation sequence, the model was trained to determine the spatial relation of one puzzle piece to another. This modification caused the model to be confronted with only two puzzle pieces, delivering better results.

In this section, the choice and performance of the applied methods were compared against state-of-the-art approaches. A broad selection of methods was carefully evaluated using sufficient training data with cross-validation. However, the results did not reach the levels achieved by state-of-the-art methods. The performance of the methods was improved by tuning the respective parameters to explore each method’s full capacity. Although this work provides insights into the applicability of the chosen methods, it may have overlooked well-performing methods outside of the scope of this study. A broader search, with less parameter-tuning and a higher number of distinctive methods, could reveal better-performing approaches for this use case. Additionally, addressing the overfitting of models is crucial for improving their performance.

### 4.2. Discussion of Models

The results presented in this work did not fully reach state-of-the-art levels. In the previous section, method implementation was discussed, but the model choice should also be considered. The implemented models were inspired by the state of the art. However, certain choices were excluded.

This work focused exclusively on classification models and did not consider regression, as proposed in other studies [25]. For example, the authors reported an AUC-ROC of up to 0.88 using certain image features and L2-regularized SVM regression. Moreover, the classification performance could be improved by using model ensembles instead of single models [29]. Ensemble learning methods achieved an accuracy, precision, and recall of 0.8097, 0.7669, and 0.7732, respectively, by using ensembles of SVM or MLP classifiers. None of the classical machine learning approaches presented in this work returned comparable scores on the test partition. However, using deep learning models, scores comparable to those presented in [29] were achieved when running the models on lesion segmentations. Three different architectures were used for CNN-based classification in this study: ConvNet, ResNet, and ConvNeXt. Multiscale approaches using the Xmas-Net architecture reportedly achieved high scores for the classification of csPCa vs. non-csPCa [27]. Xmas-Net is a modified ConvNet that extracts information from different ROI sizes of the same region.

The best performance in this work was achieved by using a pretrained model. Using self-supervised pretraining should be investigated further in future work. Further improvement could also be achieved by using larger pretrained models, such as the Segment Anything Model developed by Meta AI or the Inception V3 model from Google.

## 5. Conclusions

Altogether, the extensive evaluation of different methods and models provided an enhancement in overall performance. The pipeline performance increased by filtering out images that previously caused false-positive predictions. As the performance increase was slight, the outcome of this work indicates a direction for further research to improve medical pipeline products. It was shown that pretraining models in a self-supervised manner delivered reasonable results in the evaluated setup. The results revealed that providing models with the best available localization is beneficial, as even strong classifiers could not perform well in a pipeline with mediocre localization capabilities.

The incorporation of lesion segmentation data as localization positively impacted classification performance, addressing the first main question stated in the Introduction section. The pretraining approach based on the Genesis model improved the pipeline specificity by 0.0225, from 0.4727 to 0.4952, while maintaining the sensitivity at 0.6170, thus lowering the false positive rate. This indicated that the CNN-based approaches were more beneficial in this setup compared to the classical machine learning approaches, thereby addressing the second and third questions. It is recommended that future work focuses on the use of self-supervised or pretrained models. Investigating an end-to-end pipeline based on nn-UNet or similar models for simultaneous or staggered localization and classification could lead to further improvements.

The best-performing classifier could technically be integrated into existing pipeline products. However, given the slight performance increase on one specific dataset, it is uncertain whether products would perform similarly on new data. The small performance increase does not justify the additional time that is required to compute the image-level labels from the sliding windows. For changes to significantly impact existing medical products, higher pipeline performance would be necessary. The standalone lesion classification performance would need to translate directly into the pipeline performance. A robust and reliable image classifier could save time and resources by minimizing unnecessary inferences of segmentation models for benign prostate scans.

## Figures and Tables

**Table 1:** Metric measurements for the whole-image classification using classical ML on the test partition with the best feature configuration, respectively. Values written in bold indicate the best performance in each class.

Model	AUC-ROC	Sensitivity	Specificity	Feature Set	Selection (# Features)
SVM	**0.6016**	**0.3611**	0.8421	LBP + HOG	PCA (95)
XGB	0.5782	0.2223	**0.9342**	LBP + HOG + Haralick	None (891)
MLP	0.5762	0.3056	0.8158	LBP + HOG + Haralick	None (891)

**Table 2:** Metric measurements for the whole-image classification using CNNs on the test partition with the best feature configuration. Values written in bold indicate “the best of class”.

Architecture	AUC-ROC	Sensitivity	Specificity
ConvNet	0.4808	0.4172	0.5808
ConvNeXt	0.5038	0.3659	0.6353
ResNet	0.4958	**0.4832**	0.5159
Convolutional autoencoder (Softmax classifier)	**0.6553**	0.4722	0.7763
ConvNeXt (with Jigsaw Puzzle Pretraining)	0.5461	0.1500	**0.9105**
ConvNet (Encoding branch from Models Genesis)	0.5946	0.3611	0.6895

**Table 3:** Metric measurements for different regional inputs on the test partition. First four rows: classical ML methods, last four rows: CNN-based methods. Values written in bold indicate the best performance in each class.

Region	Model	AUC-ROC	Sensitivity	Specificity
Gland	XGB	0.5972	**0.6111**	0.5833
PZ (on ADC)	XGB	0.5467	0.4483	0.6452
Lesion	XGB	**0.6897**	0.5333	0.8462
Sliding Window	XGB	0.5291	0.1974	**0.8608**
Gland	Genesis	0.7192	0.6318	0.6578
PZ (on ADC)	Genesis	0.6393	0.5982	0.6502
Lesion	Genesis	**0.7343**	**0.7656**	0.7300
Sliding Window	Genesis	0.6598	0.5805	**0.8004**

**Table 4:** Performance measurements for each region approach as filter upstream to the lesion segmentation pipeline, listing only the best result for each region. Values written in bold indicate the best performance in each class.

Model	Region	Sensitivity	Specificity
Baseline UNet	Lesion	0.6170	0.4727
ConvNeXt	While Image	0.4231	0.4198
SVM	Gland	0.5106	0.4815
SVM	Zone	0.4894	0.4561
Genesis	Lesion	0.4808	0.3551
Genesis	Sliding window	**0.6170**	**0.4952**

## Data Availability

The data used in this study are not available due to ethical, legal, and privacy protection of the involved study subjects and to be compliant with national and international legislations of data privacy related to healthcare data for research purposes.
